# AML alters bone marrow stromal cell osteogenic commitment via Notch signaling

**DOI:** 10.3389/fimmu.2023.1320497

**Published:** 2023-12-04

**Authors:** Chiara Tomasoni, Corinne Arsuffi, Samantha Donsante, Alessandro Corsi, Mara Riminucci, Andrea Biondi, Alice Pievani, Marta Serafini

**Affiliations:** ^1^ Tettamanti Center, Fondazione Istituto Ricovero e Cura a Carattere Scientifico (IRCCS) San Gerardo dei Tintori, Monza, Italy; ^2^ Department of Molecular Medicine, Sapienza University, Rome, Italy; ^3^ Department of Medicine and Surgery, University of Milano-Bicocca, Monza, Italy; ^4^ Pediatrics, Fondazione Istituto Ricovero e Cura a Carattere Scientifico (IRCCS) San Gerardo dei Tintori, Monza, Italy

**Keywords:** acute myeloid leukemia, BMSCs (bone marrow stromal cells), AML niche, osteogenesis, Notch signaling

## Abstract

**Introduction:**

Acute myeloid leukemia (AML) is a highly heterogeneous malignancy caused by various genetic alterations and characterized by the accumulation of immature myeloid blasts in the bone marrow (BM). This abnormal growth of AML cells disrupts normal hematopoiesis and alters the BM microenvironment components, establishing a niche supportive of leukemogenesis. Bone marrow stromal cells (BMSCs) play a pivotal role in giving rise to essential elements of the BM niche, including adipocytes and osteogenic cells. Animal models have shown that the BM microenvironment is significantly remodeled by AML cells, which skew BMSCs toward an ineffective osteogenic differentiation with an accumulation of osteoprogenitors. However, little is known about the mechanisms by which AML cells affect osteogenesis.

**Methods:**

We studied the effect of AML cells on the osteogenic commitment of normal BMSCs, using a 2D co-culture system.

**Results:**

We found that AML cell lines and primary blasts, but not normal hematopoietic CD34^+^ cells, induced in BMSCs an ineffective osteogenic commitment, with an increase of the early-osteogenic marker tissue non-specific alkaline phosphatase (TNAP) in the absence of the late-osteogenic gene up-regulation. Moreover, the direct interaction of AML cells and BMSCs was indispensable in influencing osteogenic differentiation. Mechanistic studies identified a role for AML-mediated Notch activation in BMSCs contributing to their ineffective osteogenic commitment. Inhibition of Notch using a γ-secretase inhibitor strongly influenced Notch signaling in BMSCs and abrogated the AML-induced TNAP up-regulation.

**Discussion:**

Together, our data support the hypothesis that AML infiltration produces a leukemia-supportive pre-osteoblast-rich niche in the BM, which can be partially ascribed to AML-induced activation of Notch signaling in BMSCs.

## 1 Introduction

Acute myeloid leukemia (AML) is a heterogeneous clonal hematopoietic malignancy characterized by proliferation and accumulation of transformed immature myeloid precursors in the bone marrow (BM) which, competing with hematopoietic stem cells (HSCs) for nourishment and niche occupancy, lead to impaired production of normal blood cells (1). Progressive leukemia infiltration disrupts the normal BM niche and creates an abnormal microenvironment supporting AML stem cells that represent the source of AML resurgence (2, 3).

One of the key components of the BM niche is represented by bone marrow stromal cells (BMSCs), heterogeneous multipotent progenitors that can differentiate toward different cell types, such as adipocytes and osteoblasts, and that play a pivotal role in regulating HSC maintenance and differentiation. The proportion and function of BMSCs are aberrant in AML BM, creating conditions that suppress normal HSCs and preferentially support the outgrowth of AML cells. Increasing evidence revealed the existence of a reciprocal pattern of interactions between AML cells and BMSCs. Indeed, BMSCs influence various biological processes in AML, including metabolic changes and alterations in the expression of pro-survival factors, thereby conferring a growth advantage for AML cells and contributing to drug resistance (4–6). On the other side, AML cells reprogram BMSC features through different mechanisms, such as soluble factors or cell-to-cell interactions, inducing methylation aberrancies, decreased ability to provide critical niche factors, and differentiation alterations that ultimately promote AML progression (7–11). Additionally, AML-derived BMSCs exhibit a significant delay in osteogenic differentiation (12). Similar alterations were observed also in murine AML models which revealed a reshaping in BM architecture, with loss of mature osteoblasts and accumulation of osteoprogenitors (7, 13, 14). Furthermore, we previously showed that AML patient-derived BMSCs, even once removed from their pathological environment, exhibit *in vivo* reduced osteogenic differentiation capability and generate a supportive osteoprogenitor-enriched BM niche (15). Moreover, there is evidence that AML cells actively induce the generation of altered osteoblasts, through inflammatory mediators such as CCL3 and kynurenine or exosomes, to inhibit their ability to maintain normal HSCs, but effectively support leukemic cells (14, 16, 17).

However, the mechanisms that mediated the AML-osteoprogenitor cell communication, the molecular events by which leukemia affects osteogenesis, and whether this crosstalk could be harnessed for a therapeutic purpose remain largely unexplored.

Recent studies have focused on the role of Notch and Wnt signaling in the crosstalk between AML cells and BMSCs (18–20). Notch signaling is involved in BMSC-mediated support of AML cell growth and protection against chemotherapy-induced apoptosis (19, 20). Moreover, Notch signaling alterations within BM stromal cells can autonomously initiate myeloid malignancies in murine models (21). Notch signaling plays a key role also in the regulation of BMSC differentiation as well as in osteoblastogenesis and bone homeostasis (22), and the enhancement of its activation in BMSCs has already been related to an accumulation of immature pre-osteoblasts (23).

In this study, we analyzed the effect of AML cells on the osteogenic differentiation potential of BMSCs. Specifically, BMSCs exposed to AML cells acquired defects in osteogenesis related to AML-mediated activation of the Notch pathway.

## 2 Materials and methods

### 2.1 Cell lines and AML primary cells

KG-1, THP-1, U-937, HL-60 (AML cell lines) and NALM-6 and 697 (ALL cell lines) have been obtained from American Type Culture Collection (ATCC). The KG-1 and U-937 cell lines were maintained in culture with RPMI 1640 medium (EuroClone) supplemented with 10% heat-inactivated fetal bovine serum (FBS, Gibco), 2 mM L-glutamine (Invitrogen), 25 IU/ml of penicillin and 25 mg/ml of streptomycin (Invitrogen). The THP-1, NALM-6, and HL-60 cell lines were maintained in culture with 10% FBS Advanced RPMI 1640 complete medium (Gibco). The 697 cell line was maintained in culture with 20% FBS Advanced RPMI 1640 complete medium.

Primary AML cells were obtained from peripheral blood (PB) or BM samples collected from newly diagnosed AML patients. Briefly, PB- or BM-mononuclear cells (MNCs) were isolated using a Ficoll-Paque™ Plus (GE Healthcare) density gradient separation.

### 2.2 Isolation and culture of BMSCs

BMSCs were isolated from BM aspirates of healthy donors undergoing BM harvest for allogeneic transplantation. Each BM sample was centrifugated over a Ficoll-Paque density gradient to separate MNCs. BM-MNCs were seeded at a density of 2 × 10^5^ cells/cm^2^ in DMEM–low glucose medium (1 g/L, Invitrogen) supplemented with 10% FBS, 2 mM L-glutamine, 25 IU/ml of penicillin and 25 mg/ml of streptomycin. Non-adherent cells were removed 24-48 hours after initial plating by washing with phosphate-buffered saline (PBS, Euroclone). The cultures were maintained in the complete medium until they reached 70-80% confluence and then detached with 0.05% Trypsin-EDTA (Invitrogen) and re-seeded at 2 x 10^3^ cells/cm^2^. BMSC cultures were characterized by assessing morphology, clonogenic potential, proliferation, immunophenotype, and tri-lineage differentiation potential (adipogenic, osteogenic, and chondrogenic differentiation), as described in Pievani et al., 2021 (15), and used for our experiments until passage 7.

### 2.3 CD34^+^ progenitor isolation

Human CD34^+^ progenitors were isolated from cord blood (CB) units. Briefly, CB-MNCs were isolated by density gradient centrifugation, followed by immunomagnetic selection using the CD34 MicroBeads kit (Miltenyi Biotec) coupled with MACS LS Columns and MidiMACS Separator (all Miltenyi Biotec), according to the manufacturer’s instructions. Purity has been determined by flow cytometry using anti-CD34 PECy7 (clone 8G12; BD Biosciences). The median purity of isolated CD34^+^ cells was 96.1% (range from 92.5% to 99.4%).

### 2.4 Induction of osteogenesis

BMSCs (2 x 10^4^ cells/cm^2^) were cultured in the osteogenic medium consisting of DMEM-low glucose, supplemented with 10% FBS, 0.1 µM dexamethasone (Sigma-Aldrich), 10 mM beta-glycerophosphate (Sigma-Aldrich), and 0.05 mM L-ascorbic acid (Sigma-Aldrich). Cell cultures were maintained for different times, as indicated in the Results.

### 2.5 Co-culture experiments

BMSCs were seeded at 2 x 10^4^ cells/cm^2^ in complete DMEM in a 12-well plate (Corning) or a 6-well plate (Corning). After reaching 70-80% confluence, typically in 24 hours, leukemia cells were added. For AML and ALL cell lines, we plated 7 x 10^5^ cells/well in a 12-well plate and 2 x 10^6^ cells/well in a 6-well plate in basal medium (complete DMEM) or osteogenic medium. For primary AML cells or healthy CD34^+^ progenitors, we plated 1.4 x 10^6^ cells/well in a 12-well plate. The times of different co-culture experiments are indicated in the Results.

For purification of BMSCs after co-culture, the adherent layer was dissociated using 0.05% Trypsin-EDTA after washing with PBS to remove non-adherent cells. Cells were magnetically separated using anti-human CD90 MicroBeads and MidiMACS Separator (all Miltenyi Biotec), according to the manufacturer’s instructions. The positive fraction was resuspended in the QIAZOL reagent (QIAGEN Inc.).

To evaluate the effect of cell-cell contact, the co-cultures were performed using a transwell system (0.4 μm pores polyester membrane, Corning) with BMSCs in the bottom well and AML cells in the insert, at the same cell concentrations as reported above.

To evaluate the effects of Notch activation, BMSCs were stimulated with immobilized Recombinant human Jagged1 Fc Chimera (JAG1 5 µg/ml, R&D Systems). Notch activation was carried out for 72 hours.

To inhibit Notch pathway, DAPT (N-[N-(3,5-difluorophenacetyl)-l-alanyl]-s-phenylglycinet-butylester; Sigma-Aldrich) was added to the medium with a final concentration of 20 µM. Vehicle (DMSO) was added to the control group.

### 2.6 Flow cytometry

Multiparameter analyses of stained cell suspensions were performed on FACS CANTO II (BD Bioscience) and analyzed with FlowJo v10.4 software (Treestar).

To quantify the level of TNAP in BMSCs after co-culture with AML or ALL cell lines, primary AML cells, or healthy CD34^+^ progenitors, the adherent layer was subjected to trypsinization after washing with PBS to remove non-adherent cells. Cells were stained with TNAP-PE (clone W8B2; Biolegend) and with a fluorochrome-conjugated mAb specific to exclude residual leukemia cells. Specifically, CD33-PE-Cy7 (clone P67.6; BD Bioscience) was used to gate AML cells, CD19-PE-Cy7 (clone J3-119; Beckman Coulter) was used to gate B-ALL cells and CD34-FITC (clone 581; BD Pharmingen) was used to gate CD34^+^ progenitors.

The following antibodies were used for the analysis of BMSCs: CD90-PE (clone 5E10; eBioscience), CD73-PE (clone AD2; BD Pharmingen), CD105-PE (clone SN6; eBioscience), CD146-PE (clone P1H12; BD Pharmingen), CD45-FITC (clone HI30, BD Pharmingen), and CD34-FITC.

Median fluorescence intensities were calculated in co-cultured BMSCs in comparison to control with FlowJo software.

### 2.7 Gene expression analysis by quantitative real-time RT-PCR

RNA was extracted from purified BMSCs with QIAZOL reagent (Qiagen) according to manufacturer’s instructions. RNA was extracted from cell lines or from AML diagnostic samples with Trizol reagent (Applied Biosystem) following manufacturer’s instructions. One μg of total RNA was retro-transcribed using the SuperScript II Reverse Transcriptase (Invitrogen, Thermo Fisher Scientific), in the presence of random hexamers (Invitrogen). Real-time PCR was performed in an ABI 7900 real-time PCR system (Applied Biosystems), using TaqMan Gene Expression Master Mix and TaqMan probes reported in **Supplementary Table 1** (Roche Diagnostics). Data were normalized to the glyceraldehyde 3-phosphate dehydrogenase (GAPDH) gene expression. The relative mRNA expression was calculated by the comparative threshold cycle (Ct) method. The results are expressed as 2^‐ΔCt^ or fold increase as indicated in the graphs.

### 2.8 Statistical analysis

Results are shown as means ± SEM. Pairwise comparisons were performed by means of *t*-test, paired or not, depending on the experimental context. The tests were performed two-sided and the significance level was set as 0.05. Graphs and statistical analyses were produced with the GraphPad Prism 9 Software (GraphPad Software Inc).

## 3 Results

### 3.1 AML cells prime normal BMSCs towards early osteogenic phenotype by direct cell-cell contact

**Figure 1:**
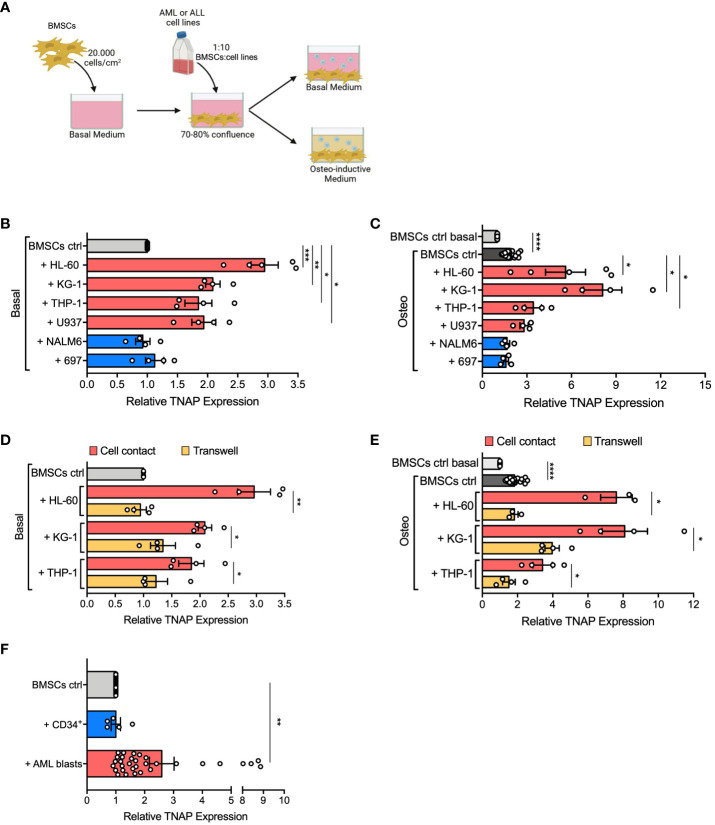
AML cells induce TNAP over-expression on BMSCs in a contact-dependent manner. **(A)** Schematic representation of *in vitro* 2D co-culture system. 7x10^4^ BMSCs were cultured in 12-well plates with AML (HL-60, KG-1, THP-1, U-937; red bars) or ALL (697, NALM-6; blue bars) cell lines in basal or osteo-inductive medium at a ratio of 1:10 (BMSCs:cell line) for 72 hours. Created with BioRender.com. **(B, C)** Relative TNAP surface expression on BMSCs assessed by flow cytometry analysis after co-culture in basal **(B)** or osteo-inductive **(C)** conditions. For HL-60: n=5 independent experiments; for THP-1, KG-1, U-937, 697, and NALM-6: n=4 independent experiments. BMSCs from 6 different donors were used for these experiments. **(D, E)** Relative TNAP surface expression on BMSCs after co-culture with AML cell lines, in direct-contact or transwell, in basal **(D)** or osteo-inductive **(E)** conditions. For THP-1 and KG-1: n=4 independent experiments using BMSCs from 4 different donors; for HL-60: n=3 independent experiments using BMSCs from 3 different donors. **(F)** Relative TNAP surface expression on BMSCs after co-culture for 72 hours with AML primary blasts or CB-derived CD34^+^ cells in basal conditions at a ratio of 1:20 (BMSCs:blasts/CD34^+^ cells). For CD34^+^: n= 5 independent experiments using 3 different BMSC strains and CD34^+^ cells isolated from 5 different CB units; for AML blasts: n=32 independent experiments using 10 different BMSC lines and AML blasts from 32 different patients. Data are presented as individual values and the mean ± SEM. ****p < 0.0001, ***p < 0.001, **p < 0.01, *p < 0.05, by paired *t*-test.

We previously demonstrated that BMSCs derived from pediatric AML patients present a significant impairment in mature bone formation *in vivo (* 15). To understand if alterations of BMSC osteogenic commitment can be specifically caused by AML infiltration in healthy BM, we evaluated the expression of tissue non-specific alkaline phosphatase (TNAP), an early osteogenic marker upregulated on AML-derived BMSCs (24), on normal BMSCs co-cultured with different AML cell lines (HL-60, KG-1, THP-1, U-937) in an *in vitro* 2D system, as described in **Figure 1A**. After 24, 48, and 72 hours of co-culture, TNAP expression on the cell surface of BMSCs was assessed by flow cytometry. AML cells, differently from ALL cells (NALM-6, 697), induced in BMSCs a progressive increase of TNAP expression, which reached significance after 72 hours (**Supplementary Figure 1A**). Notably, other BMSC surface markers were not significantly altered by exposure to AML cells (**Supplementary Figure 1B**). After 72 hours of co-culture with all the tested AML cell lines, TNAP resulted up-regulated in BMSCs both in basal (relative TNAP expression of co-cultured *vs* BMSCs alone: + HL-60, p=0.001; + KG-1, p=0.0028; + THP-1, p=0.032; + U-937, p=0.0178) and even more in osteoinductive conditions (relative TNAP expression of co-cultured *vs* BMSCs alone: + HL-60, p=0.0375; + KG-1, p=0.0138; + THP-1, p=0.0377) (**Figures 1B, C**). As expected, the osteogenic culture conditions increased *per se* the level of TNAP on BMSCs (p<0.0001) (**Figure 1C**). Conversely, ALL cell lines did not affect TNAP expression in BMSCs, neither in basal nor in osteo-inductive conditions (**Figures 1B, C**).

To understand if TNAP up-regulation on BMSCs mediated by AML depends on cell-cell contact or soluble factors, we used a transwell co-culture system. Of note, only AML in direct cell contact increased TNAP expression in BMSCs (direct contact *vs* transwell in basal conditions: + HL-60, p=0.0023; + KG-1, p=0.0153; + THP-1, p=0.01; direct contact *vs* transwell in osteoinductive conditions: + HL-60, p=0.0177; + KG-1, p=0.0265; + THP-1, p=0.0373) (**Figures 1D, E**). Moreover, the AML-conditioned medium did not affect TNAP expression in BMSCs, confirming the importance of cell-cell interaction (data not shown).

Furthermore, we evaluated if primary blasts from AML patients at the onset can prime BMSCs to osteogenic differentiation. Interestingly, co-culture with primary AML cells lead to a significant up-regulation of TNAP expression in BMSCs (p=0.0011) (**Figure 1F**). Of note, only 7 of 32 (22%) different AML samples tested did not cause any change in TNAP expression on BMSCs. However, no correlation was observed between TNAP up-regulation and genetic or morphologic-specific AML subtypes. On the contrary, co-culture with CB-derived normal CD34^+^ cells did not affect TNAP surface levels in BMSCs (**Figure 1F**).

These data demonstrate that AML cells prime osteogenic commitment of BMSCs, as highlighted by the upregulation of the early-osteogenic marker TNAP, in a contact-dependent manner.

### 3.2 AML cells block the osteogenic differentiation potential of BMSCs and alter their capacity to support normal hematopoiesis

**Figure 2:**
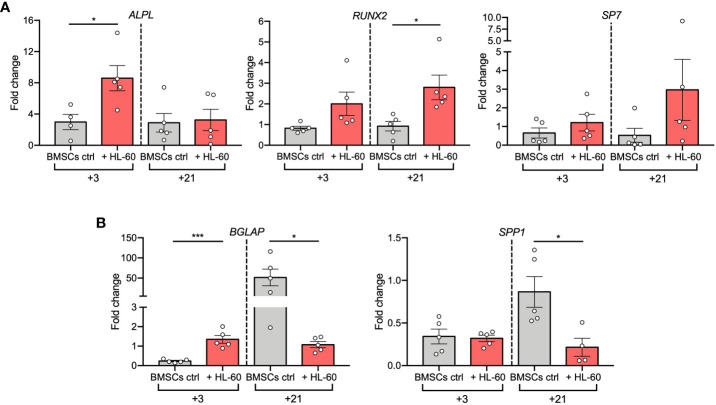
AML cell line HL-60 alters osteoblast-related gene expression in BMSCs during differentiation**. (A, B)** qRT-PCR analysis of early- **(A)** and late **(B)** -osteogenic factors performed on BMSCs alone or co-cultured with HL-60 in osteogenic conditions at the indicated time points. N≧4 independent experiments using BMSCs from 5 different donors. Data are expressed as fold change respect to control (w or w/o HL-60 in basal medium) and presented as individual values and mean ± SEM. ***p < 0.001, *p < 0.05, by paired *t* test.

To deeply investigate AML-induced alterations in the osteogenic differentiation of BMSCs, we evaluated expression changes of osteogenesis-related genes in BMSCs co-cultured with HL-60 AML cell line at 3 and 21 days after osteogenic induction. Hence, we quantified mRNA levels of early-osteogenic markers (alkaline phosphatase/*ALPL*, osterix/*SP7*, and RUNX Family Transcription Factor 2/*RUNX2*) and late-osteogenic markers (osteopontin/*SPP1* and osteocalcin/*BGLAP*). As expected, we found that levels of *ALPL*, the gene encoding for TNAP, were significantly up-regulated after 3 days of osteogenic differentiation in the presence of HL-60 cells (p=0.0275), confirming the results previously obtained by flow cytometric analysis (**Figure 2A**); however, its expression was not modulated during the 21-days of osteogenic induction and remained similar at both time points. On the contrary, BMSCs alone significantly upregulated *ALPL* expression only after 21 days of osteogenic induction. Afterwards, under osteogenic differentiation, we observed an overall higher expression of *RUNX2* in BMSCs co-cultured with HL-60 compared to control (at day 3: p=0.0233) (**Figure 2A**). No significant differences were found between BMSCs co-cultured or not with HL-60 in *SP7* expressions levels throughout the differentiation experiment (**Figure 2A**).

Analysis of expression of the osteoblast late markers *BGLAP* and *SPP1* showed a strong downregulation in BMSCs co-cultured with HL-60 compared to control at day 21 of differentiation (*BGLAP* at day 21: p=0.0160; *SPP1* at day 21: p=0.0242), suggesting an impairment in the capacity of BMSCs to differentiate into mature osteoblasts in the presence of AML cells (**Figure 2B**). Overall, these data suggest that AML cells impair osteogenic differentiation of BMSCs at a pre-osteoblastic stage.

**Figure 3:**
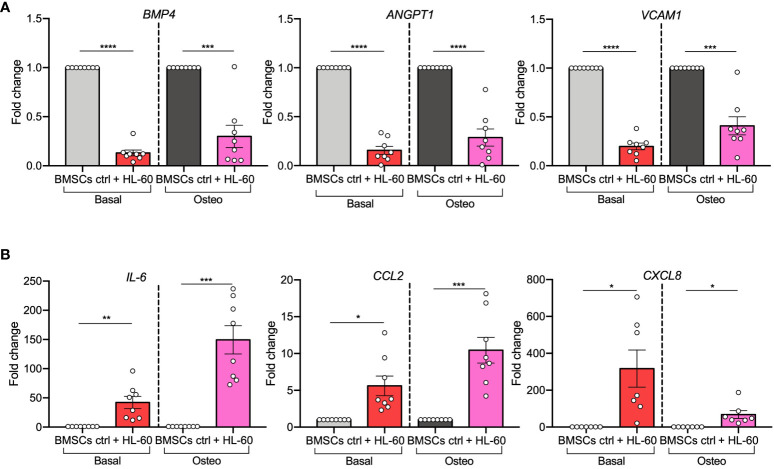
AML cell line HL-60 alters the expression of HSC-regulating and leukemogenesis-supporting genes in BMSCs and their pre-osteoblast progeny. **(A, B)** qRT-PCR analysis of HSC-supporting factors (*BMP4*, *ANGPT1*, and *VCAM1; panel A*) and leukemogenesis-supporting factors (*IL-6*, *CCL2*, and *CXCL8; panel B*) performed on BMSCs alone or co-cultured with HL-60, in basal or osteogenic conditions for 72 hours. N=8 independent experiments using BMSCs from 8 different donors. For *CXCL8*: n=7 independent experiments using BMSCs from 7 different donors. Data are expressed as fold change respect to control and presented as individual values and mean ± SEM. ****p < 0.0001, ***p < 0.001, **p < 0.01, *p < 0.05, by paired *t*-test.

Next, as it was reported that pre-osteoblasts can support leukemic cells at the expense of normal hematopoiesis, we focused on AML-induced changes in expression levels of hematopoietic-related genes. HSC-regulating genes (*BMP4*, *ANGPT1*, and *VCAM1*) *(* 25–27) were strongly down-regulated in BMSCs (*BMP4*, p<0.0001; *ANGPT1*, p<0.0001; *VCAM1*, p<0.0001) and in their pre-osteoblast progeny (*BMP4*, p=0.0005; *ANGPT1*, p<0.0001; *VCAM1*, p=0.0004) after exposure to HL-60 AML cells (**Figure 3A**). On the contrary, genes implicated in leukemogenesis (*IL-6*, *CCL2*, and *CXCL8*) *(* 28–30) were significantly increased in AML-exposed BMSCs in both basal (*IL-6*, p=0.0055; *CXCL2*, p=0.0105; *CXCL8*, p=0.0201) and osteogenic conditions (*IL-6*, p=0.0005; *CCL2*, p=0.001; *CXCL8*, p=0.0205) (**Figure 3B**). These findings support the hypothesis that AML cells influence osteogenesis-committed BMSCs contributing to the generation of a tumor microenvironment that becomes permissive to leukemia growth, disrupting normal hematopoiesis.

### 3.3 Notch signaling is involved in the crosstalk between AML cells and BMSCs

Given the fact that AML cells interfere in the early stages of BMSC osteogenic cell lineage differentiation through direct cell contact, we next aimed to identify the underlying mechanisms. Since Notch signaling is involved in the interaction between AML cells and BMSCs (19, 31) and it plays a pivotal role in the regulation of osteogenic cell lineage differentiation (23), we explored if Notch pathway relates to the altered osteogenic capacity of BMSCs in presence of AML cells.

**Figure 4:**
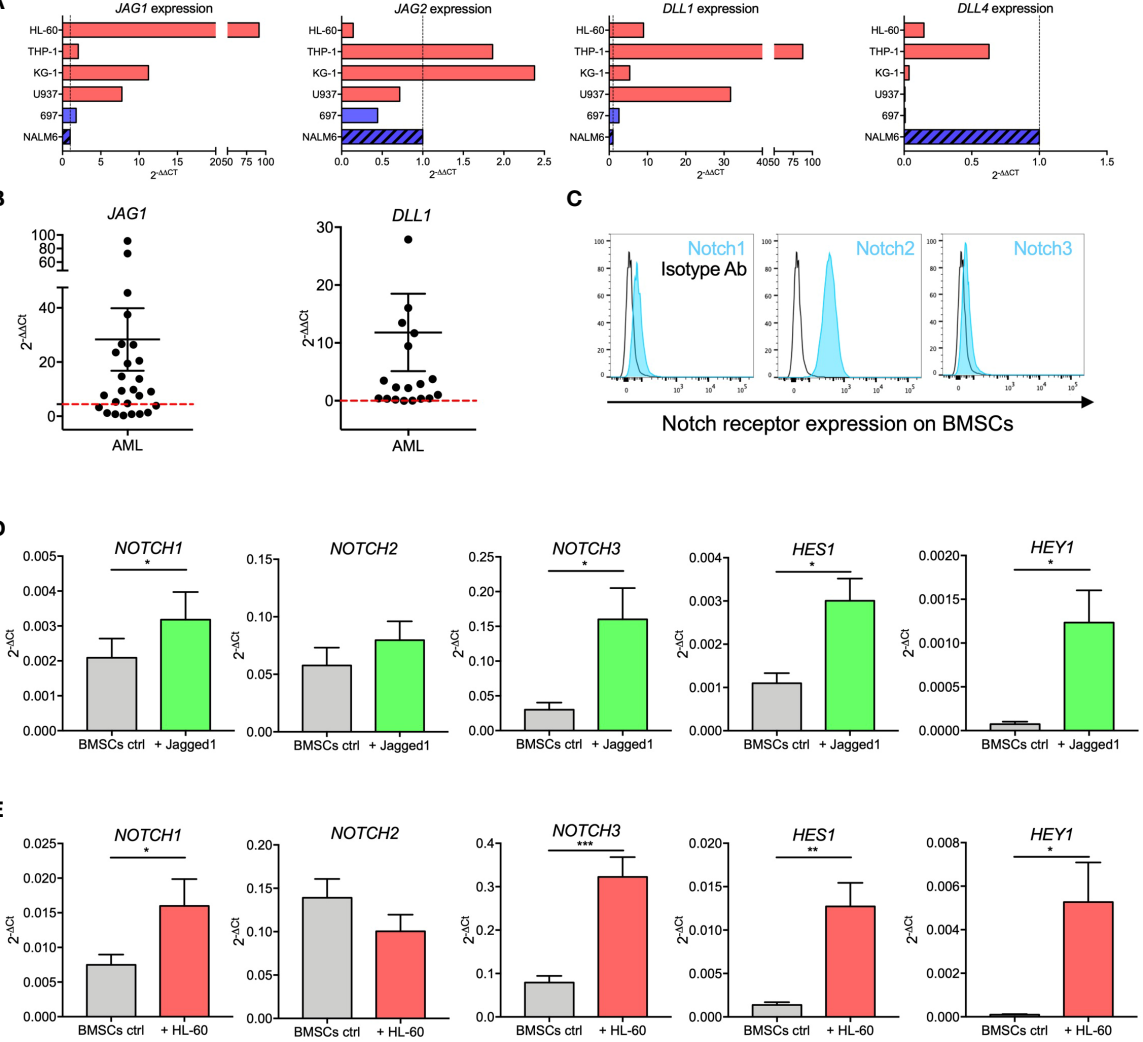
Notch signaling in BMSCs-AML crosstalk. **(A)** qRT-PCR analysis of *JAG1*, *JAG2*, *DLL1*, and *DLL4* in AML cell lines (HL-60, THP-1, KG-1, and U-937; red bars) and ALL cell line (697 and NALM-6; blue bars). On the X axis, the expression is shown as fold change, calculated as 2^-ΔΔCt^ using NALM-6 ALL cell line as reference. **(B)** qRT-PCR analysis of *JAG1* and *DLL1* in primary AML blasts. mRNA expression levels are shown as fold change, calculated as 2^-ΔΔCt^ using NALM-6 ALL cell line as reference. Each dot represents a single patient (n=26 for *JAG1*, n=19 for *DLL1*). Red dotted lines represent the median value from healthy BM. Mean and SEM were reported. **(C)** Representative histograms showing the basal expression of Notch receptors on the surface of BMSCs evaluated by flow cytometry. **(D)** qRT-PCR analysis of mRNA expression of *NOTCH1*, *NOTCH2*, *NOTCH3* and their transcriptional targets *HES1* and *HEY1* on BMSCs stimulated with immobilized recombinant Jagged1 for 72 hours. Data are expressed as 2^-ΔCt^ and presented as mean ± SEM from 7 independent experiments using BMSCs from 7 different donors. **(E)** qRT-PCR analysis of mRNA expression of *NOTCH1*, *NOTCH2*, *NOTCH3* and their transcriptional targets *HES1* and *HEY1* on BMSCs cultured alone or together with HL-60 cells for 72 hours. Data are expressed as 2^-ΔCt^ and presented as mean ± SEM from 8 independent experiments using BMSCs from 8 different donors. ***p < 0.001, **p < 0.01, *p < 0.05, by paired *t*-test.

We evaluated the expression of main Notch ligands *JAG1*, *JAG2*, *DLL1*, and *DLL4* in AML cell lines, AML diagnostic samples, and ALL cell lines. We observed that *JAG1* and *DLL1* were highly expressed in AML cell lines (**Figure 4A**). Supported by this evidence, we further confirmed *JAG1* and *DLL1* expression in diagnostic samples from AML patients, with a wide range of expression (**Figure 4B**). Moreover, Notch receptors were expressed by BMSCs (**Figure 4C**).

We then assessed the activity of the Notch pathway in BMSCs by measuring mRNA levels of Notch receptors and their transcriptional targets after stimulation with immobilized recombinant Jagged1. Jagged1 stimulation induced a significant increase of *NOTCH1* (p=0.0183) and *NOTCH3* (p=0.0128) receptors, as well as of their transcriptional targets *HES1* (p=0.0105) and *HEY1* (p=0.0169) (**Figure 4D**). To assess if Notch signaling is involved in the crosstalk between AML and BMSCs cells, we investigated if the expression levels of Notch receptors and related transcription factors in BMSCs changed when cocultured with HL-60. Interestingly, we showed that *NOTCH1* (p=0.0249), *NOTCH3* (p=0.0005), *HES1* (p=0.0044), and *HEY1* (p=0.0242) expression was significantly increased in BMSCs co-cultured with HL-60 cells (**Figure 4E**). Similar results were also found after 72 hours of co-culture with KG-1 AML cell line (**Supplementary Figure 2**). Our findings thus corroborated the importance of Notch signaling in the crosstalk between AML and BMSCs.

### 3.4 AML-mediated Notch signaling activation in BMSCs is involved in alterations of osteogenesis

**Figure 5:**
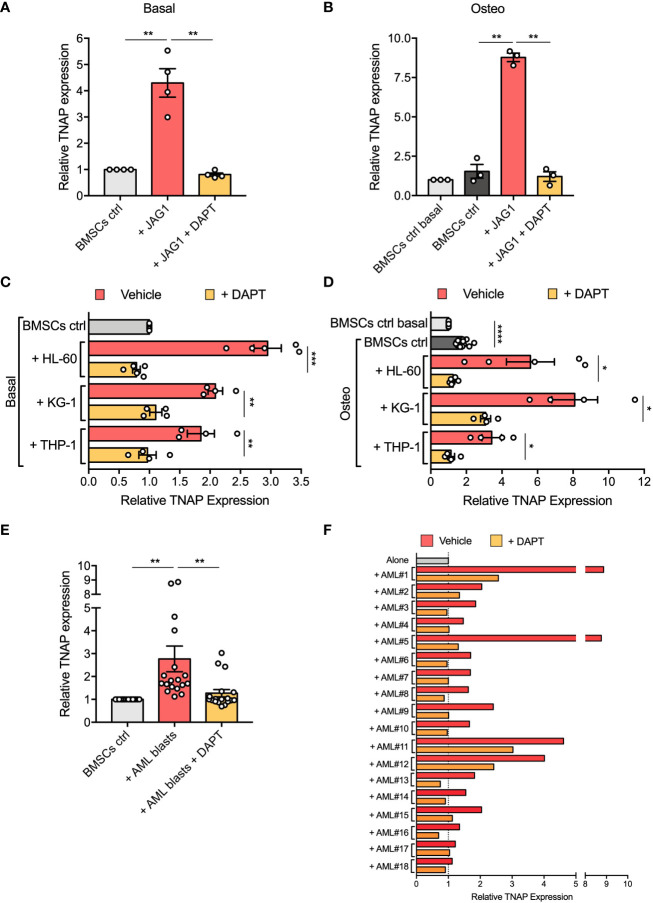
Notch signaling is involved in AML-mediated BMSC osteogenic alteration. **(A, B)** Relative TNAP surface expression on BMSCs assessed by flow cytometry analysis after 72 hours of culture in Jagged1-coated plates with or w/o DAPT, in basal or osteo-inductive conditions. For basal conditions **(A)**: n=4 independent experiments using BMSCs from 4 different donors; for osteo-inductive conditions **(B)**: n=3 independent experiments using BMSCs from 3 different donors. **(C, D)** Relative TNAP surface expression on BMSCs after 72 hours of co-culture with AML cell lines (HL-60, KG-1, THP-1) with or w/o DAPT, in basal or osteo-inductive conditions. For both basal **(C)** and osteo-inductive **(D)** conditions: n=5 independent experiments using BMSCs from 5 different donors for HL-60 cell line, n=4 independent experiments using BMSCs from 5 different donors for other cell lines. **(E, F)** Relative TNAP surface expression on BMSCs after 72 hours of co-culture with primary AML blasts with or w/o DAPT. N=18 independent experiments using 10 different BMSC lines and AML blasts from 18 different patients. Data are presented as individual values and the mean ± SEM **(E)** or as individual experiment **(F)**. ****p < 0.0001, ***p < 0.001, **p < 0.01, *p < 0.05, by paired *t*-test.

To determine the functional contribution of Notch signaling in affecting BMSCs osteogenic maturation, we first evaluated by flow cytometry the TNAP expression on the surface of BMSCs stimulated with immobilized recombinant Jagged1 for 72 hours. We showed that Jagged1 treatment significantly increases the relative TNAP expression levels in both basal (p=0.0089) and osteogenic conditions (p=0.0026) (**Figures 5A, B**). The addition of DAPT, a γ-secretase inhibitor that blocks the generation of Notch intracellular domains, prevented Notch signaling activation in BMSCs treated with Jagged1 preventing also the Jagged1-induced up-regulation of TNAP in both conditions (basal condition: p=0.0088; osteogenic condition=0.0024), proving the involvement of the Notch signaling in the regulation of TNAP expression in BMSCs (**Supplementary Figure 3** and **Figures 5A, B**).

Then, we asked if the increased expression of TNAP in BMSCs cocultured with AML cells is mediated by Notch signaling. Strikingly, treatment with DAPT successfully avoided the up-regulation of TNAP in BMSCs cocultured with AML cell lines restoring its expression to control level (DAPT *vs* vehicle in basal conditions: HL-60, p=0.0006; KG-1, p=0.0032; THP-1, p=0.0037; DAPT *vs* vehicle in osteoinductive conditions: HL-60, p=0.0305; KG-1, p=0.0164; THP-1, p=0.0155) (**Figures 5C, D**).

The same result was obtained co-culturing BMSCs with primary AML blasts (p=0.0056) (**Figure 5E**). Notably, DAPT treatment in each specific co-culture brought TNAP relative expression back to basal expression, except for AML#1, AML#11, and AML#12 in which TNAP expression after DAPT-treatment remained higher compared to control condition, but still significantly decreased compared to vehicle condition (**Figure 5F**). All together, these data suggest the involvement of Notch signaling in the boost toward osteoprogenitor of BMSCs induced by AML cells.

## 4 Discussion

In this study, we showed that AML cells alter the osteogenic differentiation potential of BMSCs and that Notch activation plays a key role in this process.

In the last decade, numerous evidences suggested that AML cells induce alterations in non-hematopoietic BM niche cell populations, affecting their capacity to regulate and support normal hematopoiesis while favoring disease progression and therapy resistance (32–34). In particular, murine models have revealed that myeloid neoplasia alters the bone tissue architecture (2, 16). Hanoun et al. reported that murine AML BM contains a higher number of osterix-expressing osteogenic cells than healthy BM (13). In line with this result, we previously showed that BMSCs derived from AML patients are skewed toward an ineffective osteogenic cell lineage differentiation with an accumulation of osteoprogenitors in an *in vivo* ossicle model (15).

However, it is not fully elucidated how AML cells alter the osteogenic commitment of BMSCs. Deeply characterizing these alterations and the associated pathways is paramount to find new targets for potential niche-targeted therapies for AML patients.

We assessed the osteogenic differentiation of normal BMSCs in the presence of AML cells. Using a 2D co-culture system in which BMSCs and blasts were maintained in direct contact both in basal and osteogenic conditions, we found that AML cells, but not normal CD34^+^ cells, induce an early up-regulation of TNAP expression on BMSC surface. Furthermore, the direct interaction between AML cells and BMSCs is necessary to increase TNAP levels, a marker of early osteoprogenitor cells (35), reported to be overexpressed on AML patient-derived BMSCs compared with normal ones (24). Under conditions that induce osteogenic differentiation, we observed overall higher expression of *RUNX2* in BMSCs in the presence of AML cells. Conversely, BMSCs co-cultured with AML cells in osteogenic conditions for 21 days showed a remarkably lower up-regulation of late osteogenesis-related markers *BGLAP* and *SPP1* compared to control.

Overall, these data indicate that AML cells prime osteogenic commitment of BMSCs in a contact-dependent manner but impair terminal osteogenic differentiation of BMSCs through the generation of osteoblast progenitors that cannot fully undergo successful complete maturation. It would be interesting to study AML-BMSCs interactions *in vivo* using human AML xenografts and humanized bone marrow ossicle models to validate our results (36).

Our observations agree with findings obtained from murine AML models which display a significant inhibition of osteogenesis and a decreased number of mature osteoblasts associated with bone volume loss (7, 16, 37). Despite these animal model data, clinical observation remains conflicting.

A decreased osteogenic differentiation potential has been observed in high-risk AML-derived BMSCs (38). Notably, Chen and colleagues reported a reduced amount of the bone formation marker osteocalcin in the serum of AML patients correlating with poor survival (18).

It is known that specific subpopulations of pre-osteoblasts can protect and support the maintenance of chemotherapy-resistant AML clones (39). We found that AML cells induce in osteogenesis-committed BMSCs a reduction in the expression of HSC-regulating genes, such as *VCAM1*, *BMP4*, and *ANGPT1*, that could affect HSC retention and quiescence and deplete the normal HSC pool (25–27). Accordingly, in an AML patient-derived xenograft model, the progressive deregulation of these HSC-maintenance genes was associated with an increase in AML burden to the impairment of normal hematopoiesis (40). Furthermore, AML cells induce in osteogenesis-committed BMSCs an increased expression of leukemogenesis-associated genes *CCL2*, *CXCL8*, and *IL-6* involved in AML trafficking, proliferation, and chemoresistance (28–30). These could represent potential molecular mechanisms by which osteogenesis-committed BMSCs/osteoblasts exposed to AML blasts contribute to a massive proliferation of leukemic cells and failure of normal hematopoiesis.

Interestingly, we identified a possible contribution for Notch activation in the AML-mediated alterations of osteogenic differentiation in BMSCs. Wnt/b-catenin and Notch pathways play an important role by regulating the crosstalk between AML cells and the stromal microenvironment and inducing AML progression (18–21, 31). BMSCs can enhance Notch signaling in AML cells via Jagged1 and rescue them from chemotherapy-induced apoptosis (19, 20). However, whether Notch ligands expressed by AML cells can affect the osteogenic differentiation of BMSCs is unknown. We observed that Jagged1 and Dll1 were highly expressed in AML cell lines and primary AML blasts compared with normal BMNCs. In accordance, the same two ligands were reported elevated in the BM of patients with AML (41). Activation of Notch signaling was observed in BMSCs after co-culture with AML cells, as demonstrated by the increased expression of Notch receptors and Notch target genes *HES1* and *HEY1*. In addition, we found that the expression of TNAP was increased in BMSCs after Notch activation through immobilized recombinant Jagged1. Once γ-secretase inhibitor DAPT was added to the co-culture system, the TNAP up-regulation was prevented. Notch proteins are prominent targets of γ-secretase supporting the hypothesis that AML cells can alter the osteogenic potential of BMSCs by activating Notch signaling. However, we cannot exclude that other effects, such as the interference of other γ-secretase-dependent pathways, can play additional roles.

This implication of Notch as an important pathway to suppress functional osteoblast differentiation is not surprising. Several evidences support that activation of Notch signaling in BMSCs induces early stages of osteogenic differentiation but prevents the formation of mature functional osteoblasts (23). *In vitro* studies have shown that Notch activation in BMSCs or osteoprogenitors has a negative impact on osteogenic differentiation (42). Engin et al. observed that activation of Notch signaling *in vivo* induces the formation of bone structures with increased proliferation of immature osteoblast precursors (43). Furthermore, previous studies have already implicated the activation of Notch signaling in impaired osteogenic differentiation of BMSCs in other hematological malignancies, such as ALL (44, 45), myelodysplastic syndromes (46), and multiple myeloma (47).

Although we showed that blocking Notch activation can partially recover the dysregulated osteogenesis, this does not exclude that other molecular mechanisms can contribute to AML-specific alteration of BMSC osteogenic commitment. Soluble factors (24), exosomes (14), and other contact-dependent pathways (18) have already been implicated in this process.

In summary, our results suggested that AML cells can induce an ineffective osteogenic differentiation of BMSCs that can be implicated in the generation of a pre-osteoblast/osteoprogenitor-cell rich niche favorable for leukemia growth. Activation of the Notch signaling pathway not only induces AML cell proliferation and chemoresistance but can also be involved in the altered osteogenic differentiation of BMSCs exposed to AML cells. However, to definitely validate the implication of Notch signaling in the niche remodeling, it will be important to investigate its factors at the protein level. Furthermore, the data collected in this study support the hypothesis that Notch inhibitors might be an effective approach to target the interaction of AML cells and BMSCs and normalize osteogenesis in AML BM, which is supporting for malignant hematopoiesis (48). Inhibition of Notch signaling may inhibit AML growth and rehash the altered BM niche, representing a novel therapeutic concept in AML. However, the Notch-targeted therapy resulted to be challenging because the undesired “on-target” activity may potentially lead to significant toxicity. Further studies for better characterizing mechanisms used by AML cells to affect BMSC differentiation will increase our understanding of how blasts remodel the BM microenvironment and provide the potential for new avenues of microenvironment-directed agents for combinatorial therapy.

## Data availability statement

The original contributions presented in the study are included in the article/**Supplementary Material**. Further inquiries can be directed to the corresponding author.

## Ethics statement

The studies involving humans were approved by Ethical Committee of San Gerardo Hospital, Monza (IT). The studies were conducted in accordance with the local legislation and institutional requirements. Written informed consent for participation in this study was provided by the patients or the participants' legal guardians/next of kin.

## Author contributions

CT: Conceptualization, Data curation, Formal Analysis, Investigation, Methodology, Writing – original draft, Writing – review & editing, Visualization. CA: Writing – review & editing, Investigation. SD: Writing – review & editing, Investigation. AC: Supervision, Writing – review & editing. MR: Supervision, Writing – review & editing. AB: Writing – review & editing, Supervision. AP: Conceptualization, Data curation, Investigation, Methodology, Writing – original draft, Writing – review & editing, Formal Analysis, Project administration, Validation, Visualization. MS: Funding acquisition, Resources, Supervision, Writing – review & editing, Conceptualization, Project administration.
