# Supplementary material for: AML alters bone marrow stromal cell osteogenic commitment via Notch signaling
Source: Front Immunol. 2023 Dec 4;14:1320497. doi: 10.3389/fimmu.2023.1320497 (PMC10725948; doi:10.3389/fimmu.2023.1320497)
Supplement: Supplementary file 1 [file DataSheet_1.docx]

# Supplementary Figures and Tables

## Supplementary Figures


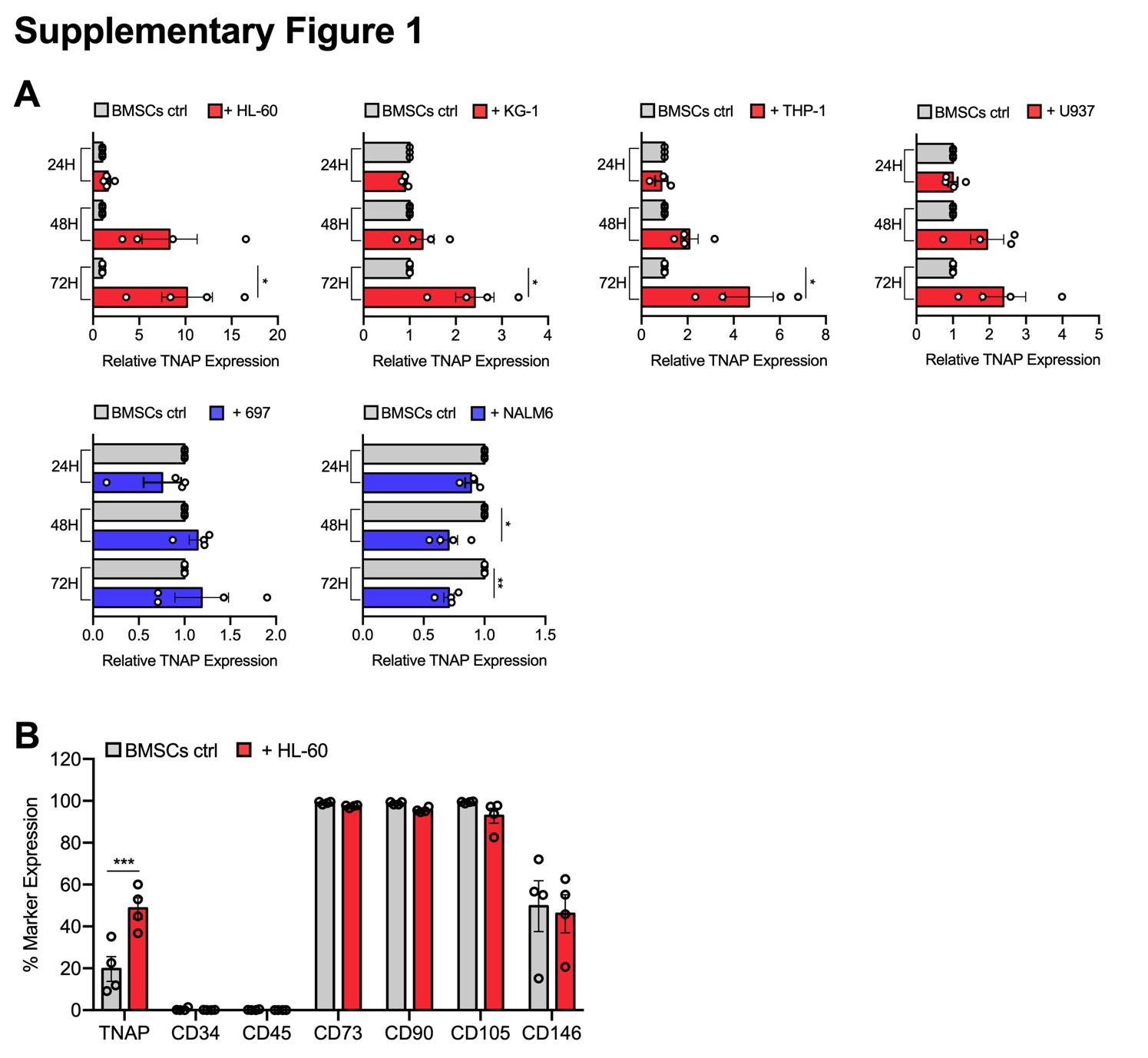


**Supplementary Figure 1.** **AML cell lines induce a progressive TNAP over-expression on BMSCs without affecting other specific surface markers.**

(A) Relative TNAP surface expression on BMSCs was assessed by flow cytometry analysis after 24, 48, and 72 hours of co-culture with AML or ALL cell lines in basal condition (complete media). N=4 independent experiments for each cell line using BMSCs derived from 6 different donors. Data are presented as individual values and the mean ± SEM. (B) Expression of BMSC surface markers (CD146, CD105, CD90, CD73, CD45, CD34) and TNAP on BMSCs assessed by flow cytometry after 72 hours of co-culture with HL-60 AML cell line. N=4 independent experiments using BMSCs from 4 different donors. Bars represent individual values and the mean ± SEM of % positive cells. ***p < 0.001, **p < 0.01, *p < 0.05, by paired *t*-test.


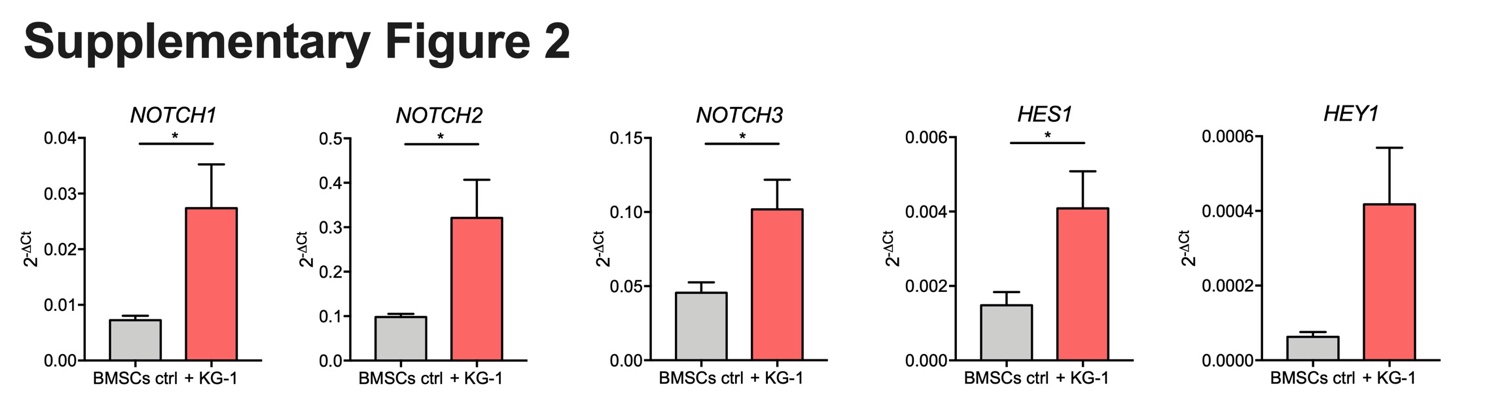


**Supplementary Figure 2. AML cell line KG-1 induces Notch signaling activation in BMSCs.**

qRT-PCR analysis of Notch signaling components performed on BMSCs in basal conditions (BMSCs ctrl) or after 72 hours of co-culture with AML cell line KG-1. N=6 independent experiments using BMSCs from 6 different donors. Data are expressed as 2^-ΔCt^ and presented as mean ± SEM. *p < 0.05, by paired *t*-test


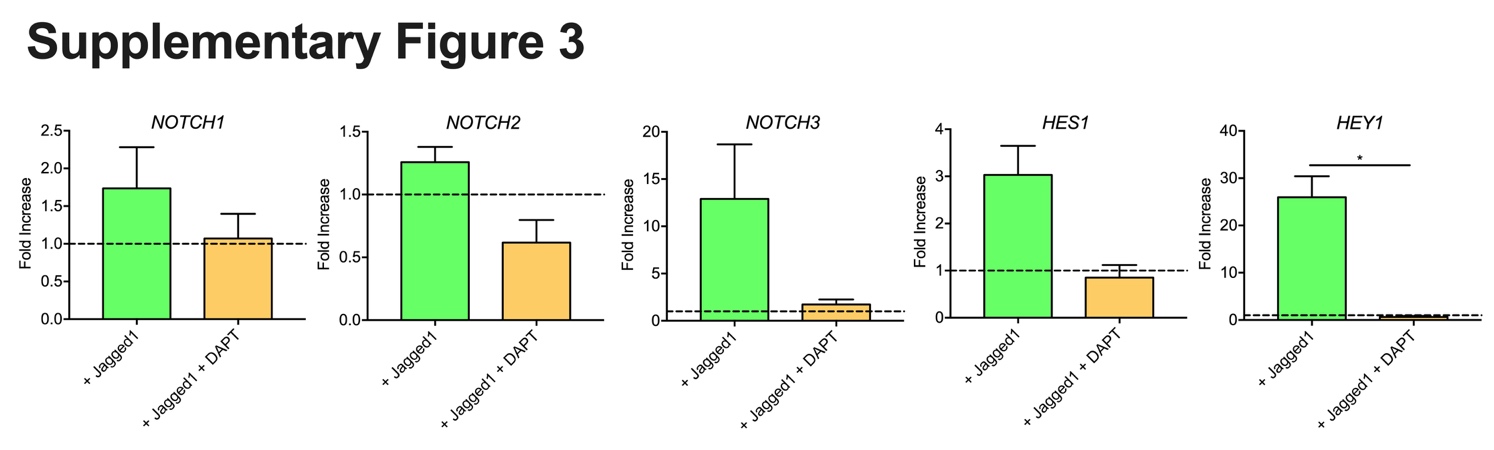


**Supplementary Figure 3. The γ-secretase inhibitor DAPT abrogates the Notch signaling activation in BMSCs.**

qRT-PCR analysis of Notch signaling components performed on BMSCs after 72 hours of stimulation with immobilized recombinant Jagged1 in presence or absence of DAPT. N=3 independent experiments using BMSCs from 3 different donors. Data are expressed as fold increase respect to control and presented as mean ± SEM. *p < 0.05, by paired *t*-test

## Supplementary Tables

| Category | Protein | Gene Symbol | Primer for RT-PCR (TaqMan assay no.) |
| --- | --- | --- | --- |
| Housekeeping | Glyceraldehyde 3-phosphate dehydrogenase | *GAPDH* | 4333764F |
| Osteogenic genes | Osteopontin  Alkaline phosphatase  Osteocalcin  Runt related transcription factor 2  Osterix | *SPP1*  *ALPL*  *BGLAP*  *RUNX2*  *SP7* | Hs00959010_m1  Hs01029144_m1  Hs00609452-g1  Hs00231692_m1  Hs01866874_s1 |
| Hematopoietic supporting genes | Vascular cell adhesion molecule 1  Angiopoietin 1  Bone morphogenetic protein 4 | *VCAM1*  *ANGPT1*  *BMP4* | Hs01003372_m1  Hs00181613_m1  Hs00370078_m1 |
| Leukemogenesis supporting genes | C-C motif chemokine ligand 2  C-X-C motif chemokine ligand 8  Interleukin 6 | *CCL2*  *CXCL8*  *IL6* | Hs00234140_m1  Hs00174103_m1  Hs00174131_m1 |
| Notch signaling genes | Jagged1  Jagged2  Delta like canonical Notch ligand 1  Delta like canonical Notch ligand 4  Notch receptor 1  Notch receptor 2  Notch receptor 3  Hes family bHLH transcription factor 1  Hes related family bHLH transcription factor with YRPW motif 1 | *JAG1*  *JAG2*  *DLL1*  *DLL4*  *NOTCH1*  *NOTCH2*  *NOTCH3*  *HES1*  *HEY1* | Hs00164982_m1  Hs00171432_m1  Hs00194509_m1  Hs00184092_m1  Hs01062014_m1  Hs01050719_m1  Hs01128541_m1  Hs00172878_m1  Hs00232618_m1 |

**Supplementary Table 1. Quantitative RT-PCR primers.**
